# Analyzing Lower Limb Muscle Imbalance Patterns Associated With Chronic Ankle Instability

**DOI:** 10.7759/cureus.77529

**Published:** 2025-01-16

**Authors:** Sonam Jagadale, Sandeep Shinde, Sawani Aphale

**Affiliations:** 1 Department of Musculoskeletal Sciences, Krishna College of Physiotherapy, Krishna Vishwa Vidyapeeth, Deemed to be University, Karad, IND

**Keywords:** functional instability, gait analysis, injury prevention, muscle strength, obesity

## Abstract

Objectives: Our main objective was to find a complex relationship between chronic ankle instability (CAI) and factors contributing to lower limb proximal-distal muscle imbalance.

Methods: This cross-sectional study, conducted in the physiotherapy department of Krishna Vishwa Vidyapeeth, Karad, included 111 volunteers selected through simple random sampling. Outcome assessments involved manual muscle testing, range of motion evaluation, and gait pattern analysis based on specific inclusion and exclusion criteria.

Results: The study interpreted that individuals with CAI exhibited significant muscle imbalances on the affected side compared to the unaffected side. Core muscle weakness was present in 97 (87.2%) of patients involving multifidus and rectus abdominals majorly, while 90 (84.6%) experienced lower limb muscle weakness. The ankle joint's range of motion was the most affected, with ankle plantar flexors and evertors showing the greatest weakness. Abnormalities in gait were also noted, with cautious gait observed in 83 (75%) patients, knee hyperextension in 24 (22%), and foot slap in four (3%).

Conclusion: The present study concluded that CAI could cause abnormal gait patterns due to weak muscle strength, impaired muscle length-tension relationships, and reduced range of motion. An efficient core gives optimal efficiency in the entire kinetic chain during movements like acceleration, deceleration, and dynamic stabilization and provides proximal stability to the lower extremities. In the study, lower limb muscle imbalance and core muscle weakness are significant contributors to CAI, impacting both stability and functional performance. Considering the study findings as an important part of patient assessment, it will be used to customize the rehabilitation programs as per the patient's requirement and, thus, enhance positive outcomes.

## Introduction

Chronic ankle instability (CAI) is a prevalent condition often resulting from recurrent ankle sprains, leading to functional limitations and the risk of further injuries being increased [1]. One contributing factor to CAI is lower limb muscle imbalance, which can compromise stability and proprioception. This analysis examines the specific muscle imbalances present in individuals with CAI, focusing on the roles of key muscle groups such as the peroneals, tibialis anterior, and gastrocnemius. These imbalances affect joint mechanics and overall lower limb function. Abnormal bilateral asymmetry (between homologous) and disruption of the agonist-antagonist ratio correlated with an imbalance in muscle strength. According to biomechanics, an agonist muscle group may contract concentrically to produce limb motion while its antagonist muscle group simultaneously creates an eccentric effort meant to decelerate and essentially protect the affected joint.

The altered relationship between the muscles that are prone to tightness and those that are more prone to weakness is known as muscular imbalance. There are numerous contractile and noncontractile components that influence how tight or loose muscles are. Imbalance is a systemic reaction involving a number of striated muscles rather than a single isolated muscle. The relationship between muscle strength and flexibility and agonist-antagonist disruption has been frequently reviewed in the literature [2].

Hamstring injuries often require the potential intervention of imbalance [3]. It demonstrated the high rate (70% of cases) of problems with the knee flexor performance in relation to past hamstring strains and persistent complaints. Chronic pain has an association with a protective adaptive response in muscles in which agonists decrease in tone while antagonists increase in tone [4].

Muscle imbalance often results from the relationship between tonic and phasic muscles. Tonic muscles, which support posture and endurance, can develop increased tone due to overuse or compensatory actions. In contrast, phasic muscles, designed for quick movements, may weaken and exhibit decreased tone when not sufficiently activated. Janda conceptualized muscle imbalance as an impaired relationship between muscles prone to tightness or shortness and muscles prone to inhibition [4].

The gait cycle is 60% phase of stance and 40% swing phase. Smooth and effective walking patterns require proper balance, timing, and muscle recruitment. CAI often leads to distinct alterations in gait patterns, primarily characterized by an unstable and cautious approach to walking. Individuals exhibit a shorter stride length and reduced push-off strength, compensating for the fear of reinjury. This causes an increased reliance on the medial side of the foot, which results in an altered center of gravity (COG) and uneven weight distribution. Additionally, these individuals might demonstrate a tendency to “limp” or adopt a more rigid ankle posture to minimize movement in the joint, further contributing to an inefficient and asymmetrical gait. Such compensatory mechanics can lead to instability and increase the risk of further injury over time. Incorrect patterns and wasteful energy expenditure are the outcomes of any imbalance or poor muscle recruitment and synchronization in any segment of the kinetic chain. Muscle imbalance may also develop from occupational or recreational activities in which there is persistent use of certain muscles without adequate exercise of opposing muscles [5]. The primary impairments associated with CAI include reduced proprioception, diminished neuromuscular control, weakened strength, and compromised postural stability [6].

Proprioception deficit has a critical role in CAI. Individuals with CAI often experience ankle sprain recurrently and persistent instability, partly due to impaired proprioception feedback. Proprioceptors in the ankle joint provide information about joint position and movement. In CAI, these receptors can be damaged or function poorly, leading to an inability to accurately sense the position of the ankle. The central nervous system is thought to get the majority of proprioception information from the muscle afferents surrounding the body's primary joints. Damage to the lateral ligament complex of the ankle joint results in proprioceptive deficits, thereby compromising the body's ability to detect joint position and movement. This impairment is believed to contribute to recurrent ankle inversion injuries and episodes of instability, commonly described as a sensation of "giving way." It was widely accepted that damage to the lateral ligament complex of the ankle joint led to proprioceptive deficits, thereby predisposing individuals to repeated inversion injuries and episodes of instability, commonly referred to as "giving way." Proprioception is measured clinically through the assessment of kinesthesia (threshold to detection of passive movement) and joint position sense [7].

The rectus abdominis and oblique abdominal muscles are engaged in a direction-specific manner during limb movements, contributing to postural support before the movement. Impaired postural control arises from a combination of proprioceptive deficits and neuromuscular dysfunction. Research shows that individuals with ankle instability exhibit compromised postural control [8]. A one-legged standing balance test was used to demonstrate deficiencies in postural control and balance [9]. More dynamic tests, like the star excursion balance test [10], time-to-stabilization, and the recently created dynamic postural stability index [11], have been used to supplement this static test. All of these tests have demonstrated postural control deficiencies in people with CAI. Compared with uninjured controls or their unaffected side, individuals with functional instability had a considerably lower reach.

Two contributing factors to CAI are mechanical ankle instability and functional ankle instability [12]. Various insufficiencies contribute to different types of instability. Mechanical insufficiencies include pathological laxity, disrupted arthrokinematics, synovial alterations, and degenerative changes. Functional insufficiencies encompass impaired proprioception, disrupted neuromuscular control, reduced strength, and compromised postural stability [10]. While mechanical and functional instability can occur independently, researchers have hypothesized that their combined effects are the most likely contributors to the development of CAI [10,12]. An acute ankle sprain injury must occur due to the development of CAI. After ankle injuries, people with CAI frequently report episodes where their ankle feels unstable [13]. CAI is often linked to localized ankle dysfunction and neuromuscular impairments, including core muscle involvement. A strong core helps maintain overall body stability, which is essential for balance during activities that stress the ankles. The core helps distribute forces throughout the body. A weak core can lead to compensatory movements that increase stress on the ankle joint. Core muscles facilitate coordinated movement patterns and help maintain stability during dynamic activities, reducing the risk of sprains.

## Materials and methods

The study's major goal was to find a complex relationship between CAI and factors that contribute to lower limb proximal to distal muscle imbalance. Also, the study may help understand that CAI includes ankle proprioception deficits, muscular weakness, impaired balance, postural control deficits, ligaments, and joint laxity. This cross-sectional study was carried out at the Krishna College of Physiotherapy, Karad. It includes 111 participants. Depending on inclusion and exclusion criteria, for this study, 111 individuals were taken using the method of simple random sampling. Subjects were included in the study: 1) age group: 20-45 years, 2) subjects willing to participate, 3) both genders, and 4) sensation of ankle instability or giving way. The subjects excluded were 1) a history of fractures or surgery, 2) pregnant women, 3) children, and 4) disabled people.

The procedure was thoroughly explained to all participants, and written informed consent was obtained from those who voluntarily chose to participate in the study. Additionally, demographic information was collected. The research was performed at Karad for one year. A total of 111 subjects were selected using simple random sampling. The individuals gave their information consent and underwent assessments including manual muscle testing (MMT) and range of motion. The results were calculated based on a detailed assessment.

Outcome measures

Manual Muscle Testing

MMT is a standardized series of evaluations designed to assess muscle strength and function. Evaluating muscle strength is a crucial component of this assessment, as it helps determine the capacity of muscles or muscle groups to perform movements and offer stability and support. MMT specifically involves the practitioner applying resistance while the patient attempts to move a muscle or group of muscles, allowing for an objective measurement of strength and identifying areas of weakness.

Range of Motion

A goniometer assessed the range of motion for ankle dorsiflexion, plantar flexion, eversion, and inversion. Before measurements, the goniometer was calibrated to zero, and the patient was instructed to move the joint through its complete range of motion. Assessment is important for determining joint flexibility, identifying limitations, and tracking progress in rehabilitation, as it reflects the overall function and mobility of the ankle joint.

Statistical analysis

Graphical presentation was done and statistical percentages were calculated with the help of Microsoft Excel (Microsoft Corporation, Redmond, WA). Participants' descriptive data are shown as percentages, and descriptive statistics were demonstrated in a table format.

## Results

Table 1 shows that among the 111 participants, 49 (44.14%) were aged between 25 and 35 years, and 62 (55.86%) participants were between 36 and 45 years. Participants were categorized according to grades of obesity. A total of 58 (52%) people were overweight and 53 (48%) people had grade 1 obesity. On the other hand, history was additionally taken into account among 111 participants: 84 (77%) had an ankle sprain, seven (6%) had flat feet, five (4%) had ankle arthritis, five (4%) had pes cavus, five (4%) had peroneal tendinitis, and five (4%) had Achilles tendinitis. The right ankle was affected in 48 individuals (43.2%), the left ankle in 34 individuals (30.6%), and both ankles were affected in 29 individuals (26.2%).

**Table 1:** Sociodemographic characteristics of participants (n = 111) BMI: body mass index

Demographic variable	Participants, n (%)
Age (years)	
25-35	49 (44.14%)
36-45	62 (55.86%)
BMI	
Overweight	58 (52%)
Grade 1 obesity	53 (48%)
Ankle affected	
Right	48 (43.2%)
Left	34 (30.6%)
Both	29 (26.2%)
History	
Ankle sprain	84 (77%)
Pes planus	7 (6%)
Ankle arthritis	5 (4%)
Pes cavus	5 (4%)
Peroneal tendinitis	5 (4%)
Achilles tendinitis	5 (4%)

Table 2 interprets that MMT of core muscles assesses strength and stability, with grades 3-5 indicating varying levels of functional capacity. Grade 3 (fair) suggests that the individual can perform against gravity actions but may struggle with resistance, reflecting potential weakness that can compromise postural stability and overall function. Grade 4 (good) indicates that the individual can withstand moderate resistance, suggesting better core control and stability, which are important for efficient movement and injury prevention. Grade 5 (normal) denotes optimal strength, allowing the individual to resist maximal resistance and maintain robust core stability during dynamic activities. The main core muscles include transversus abdominis, rectus abdominis, internal and external obliques, and multifidus. After assessing the muscles strength of these muscles, it was seen that 97 (87.2%) patients had core muscle weakness as patients were not able to perform the movements when resistance was given.

**Table 2:** MMT of core muscles MMT: manual muscle testing

Muscle group	MMT score	Participants, n (%)
Transversus abdominis	5/5	14 (12.6%)
	4/5	75 (67.5%)
	3/5	23 (20.7%)
Rectus abdominis	5/5	18 (16.2%)
	4/5	71 (64%)
	3/5	22 (19.8%)
Obliques (internal/external)	5/5	16 (14.4%)
	4/5	70 (63.1%)
	3/5	25 (22.5%)
Multifidus	5/5	15 (13.5%)
	4/5	74 (66.7%)
	3/5	27 (24.2%)

Table 3 ​interprets that MMT for ankle dorsiflexors, plantar flexors, invertors, evertors, knee flexors, and extensors is essential for evaluating muscle strength and functional capacity. Grades of 3-5 indicate varying levels of strength: grade 3 (fair) suggests limited ability to perform functional tasks against gravity; grade 4 (good) reflects the ability to withstand moderate resistance, indicating a more favorable functional capacity; while grade 5 (normal) denotes optimal muscle strength, crucial for high-demand activities like running and jumping. A total of 90 (84.6%) patients had lower limb muscle weakness as they were unable to perform the activities when resistance was applied.

**Table 3:** MMT of lower limb muscles MMT: manual muscle testing

Muscle	Grade 3 (moderate)	Grade 4 (mild weakness)	Grade 5 (normal strength)
Tibialis anterior	14 (12.6%)	60 (54%)	37 (33.3%)
Peroneal	15 (13.5%)	62 (55.8%)	34 (30.6%)
Gastrocnemius	17 (15.3%)	61 (55%)	33 (29.7%)
Soleus	15 (13.5%)	62 (55.9%)	34 (30.6%)
Quadriceps	12 (10.8%)	61 (55%)	38 (34.2%)
Hamstring	9 (11.7%)	58 (52.2%)	40 (36.03%)
Gluteus medius	8 (7.2%)	56 (50.5%)	47 (42.3%)

Cautious gait, knee hyperextension gait, and foot slap gait are common compensatory strategies observed in CAI patients, as given in Table 4. A cautious gait often indicates a protective mechanism where the individual attempts to minimize the risk of reinjury, leading to slower, more deliberate movements. Knee hyperextension gait occurs as the patient seeks stability by locking the knee during stance, which can place additional stress on surrounding structures and may contribute to further instability. Foot slap gait, characterized by a lack of dorsiflexor control during the heel contact phase, suggests weakened ankle dorsiflexors and impaired proprioception, leading to decreased control and stability upon foot placement. Collectively, these gait patterns reflect the underlying instability and functional limitations associated with CAI.

**Table 4:** Gait patterns caused due to CAI CAI: chronic ankle instability

Gait type	Total number of individuals with gait (%)	Gait characteristic	Participants, n (%)
Cautious gait	75%	Wider base of support	39 (35%)
		Slow walking speed	22 (20%)
		Shorter stride length	22 (20%)
Knee hyperextension	22%	Muscle imbalance	13 (12%)
		Fear of instability	11 (10%)
Foot slap	3%	Weakness of dorsiflexors	4 (3%)

Table 5 underlines that CAI can significantly impact the range of motion in the ankle, knee, and hip joints, creating a complex interplay that affects overall lower extremity function. Individuals with CAI often experience limited plantar flexion, dorsiflexion, inversion, and eversion in the ankle, which can lead to compensatory adjustment in the knee joint, resulting in altered kinematics. Plantar flexors and evertors are the most commonly affected, followed by hip extensors and then knee joint flexors. Consequently, these interrelated changes can disrupt gait mechanics and exacerbate instability.

**Table 5:** Range of motion of lower limbs including three joints: hip, knee, and ankle

Joint	Joint movement	Severity	Participants, n (%)
Ankle	Plantar flexion	Mild	30 (27.0%)
		Moderate	41 (37.0%)
		Severe	40 (36.0%)
	Dorsiflexion	Mild	26 (23.5%)
		Moderate	50 (45.0%)
		Severe	35 (31.5%)
	Inversion	Mild	45 (40.5%)
		Moderate	35 (31.5%)
		Severe	31 (28.0%)
	Eversion	Mild	30 (27.0%)
		Moderate	39 (35.2%)
		Severe	42 (37.8%)
Knee	Flexion	Mild	55 (49.5%)
		Moderate	28 (25.2%)
		Severe	28 (25.2%)
	Extension	Mild	60 (54.0%)
		Moderate	30 (27.0%)
		Severe	21 (18.9%)
Hip	Flexion	Mild	53 (47.7%)
		Moderate	35 (31.5%)
		Severe	23 (20.8%)
	Extension	Mild	48 (43.2%)
		Moderate	32 (28.8%)
		Severe	31 (28.0%)
	Abduction	Mild	50 (45.0%)
		Moderate	40 (36.0%)
		Severe	21 (18.9%)
	Adduction	Mild	55 (49.5%)
		Moderate	30 (27.0%)
		Severe	26 (23.5%)

## Discussion

The study aimed to investigate the lower limb muscle imbalance in CAI. CAI causes muscle imbalance in the lower limb; because of COG, the biomechanical chain easily gets affected. The study found that individuals with CAI exhibited significant muscle imbalances on the affected side compared with the unaffected side. Core muscle weakness was present in 97 (87.2%) patients involving multifidus and rectus abdominis majorly, while 90 (84.6%) experienced lower limb muscle weakness. The ankle joint's range of motion was the most affected, with ankle plantar flexors and evertors showing the greatest weakness. Abnormalities in gait were also noted, with cautious gait observed in 83 (75%) patients, knee hyperextension in 24 (22%), and foot slap in four (3%).

Ankle instability can cause gait deformity, postural imbalance, and another muscular imbalance. Also, anatomical and physiological variations between males and females can be considered a risk factor for lower limb pathologies [14]. A previously conducted study stated that some individuals found hyperextension of the knee and lateral rotation of the leg on the thigh. Weakness was usually found in instances of imbalance between the lateral and medial hamstrings, in which the medial hamstring was weak and the lateral hamstring was strong. This also decreased the strength of the eversion of the foot and plantar flexion of the ankle joint, and lateral stability of the ankle was decreased. In some individuals' weight-bearing positions, pronation with a flatness of the longitudinal arch was usually accompanied by an out-toeing of the forefoot. Excessive tension was applied to the muscles and ligaments on the medial side of the foot [15].

Hamstring muscle tightness can result in significant biomechanical alterations within the pelvis and lower limb, potentially leading to various complications [16]. The tibialis posterior and abductor hallucis were usually weak. Toe extensor muscles and the flexor muscles are prone to tightness, and foot pronation occurs. The current study results show that individuals with CAI showed a significant decrease in muscle balance control in the lower limb particularly affecting ankle, hip, and then knee joint muscles.

The study conducted by observations of balance deficits in subjects with CAI also observed decreased balance control on the injured side compared with the uninjured side with a unilateral chronic ankle sprain in gymnasts [17]. It is also possible that some of these individuals may experience bilateral balance deficits in the presence of unilateral ankle instability. In some cases, both ankles are affected. Those individuals present with functional insufficiencies including postural control deficits, proprioception deficits, and balance control problems [18].

Several researchers have addressed the muscular strength of the ankle while investigating the possible cases of CAI. Past studies have attributed the damage of ankle joint mechanoreceptors to impaired ankle proprioception, which may, in turn, cause balance control deficits [17]. For example, mechanoreceptor adaptation, corresponding to the prolonged length of ligament and capsule, may change ankle proprioception with joint laxity. Additionally, when the affected limb (i.e., unstable ankle) was the initial stance limb, participants with CAI decreased stance-side momentum possibly to lessen postural demands during the transition from double-to-single-limb stance. Altered lower extremity muscle, activation patterns have also been reported in individuals with CAI during gait [19] and when transitioning from double-to-single limb stance [20]. However, this strategy also results in postural instability during a static stance as the individual struggles to maintain balance and control without sufficient support from the ankle joint; the underlying cause of this postural instability is multifactorial weakness in the core muscles as well as in the lower limb muscles, which contributes significantly to the lack of stability. Furthermore, limited ankle joint range of motion impairs the ability to effectively stabilize the foot during stance. All these factors affect the individual’s ability to effectively stabilize the foot and maintain proper posture as well as gait [21]. The alterations in ankle positioning during gait are thought to result from a change in preprogrammed motor control likely due to impaired proprioception and a reduced ability to detect the position sense of the ankle joint.

The core muscles play a critical role in maintaining stability and facilitating efficient movement by acting as a protective corset around the trunk. Comprising the abdominal muscles, vertebrae, gluteal muscles, diaphragm, and pelvic floor, all these muscles work synergistically to generate intra-abdominal pressure, particularly through the activation of transverse abdominis, rectus abdominis, as well as oblique muscles, which provide postural stability as they activated in the direction-specific pattern concerning limb movement [22].

The study provides valuable insight into the role of core and lower limb muscle imbalances in CAI. Identifying that muscle weaknesses, particularly in the multifidus and rectus abdominis, significantly contribute to the condition, offers important information for rehabilitation strategies. Gait-related findings can assist clinicians in gaining a deeper understanding of the gait disturbances frequently associated with this condition and inform the development of targeted interventions. The findings indicate that correcting muscle imbalances in both the core and lower limbs is essential for managing CAI. In the future, rehabilitation programs should incorporate exercises to strengthen core stabilizers, such as the multifidus and rectus abdominis, while also focusing on enhancing lower limb muscle strength, particularly in the ankle plantar flexors and evertors. Such interventions may contribute to the reduction of instability and the enhancement of functional performance. The study’s findings suggest a highly individualized approach to treatment based on the specific weaknesses in core and lower limb muscles. All these factors contribute to enhancing positive outcomes, thereby improving patient’s quality of life.

The study primarily relied on MMT and basic range of motion evaluation. While these methods provided useful insights, the inclusion of advanced assessment tools, such as isokinetic dynamometry, electromyography, or 3D motion capture analysis, and force plate analysis could have offered more precise and objective assessments of muscle strength, activation patterns, and gait abnormalities. Future research aims to expand the scope of the analysis by incorporating data from various locations or regions. Additionally, future studies could benefit from including a larger sample size.

## Conclusions

The present study concluded that CAI can cause abnormal gait patterns due to weak lower limb muscle strength, impaired muscle length-tension relationships, and reduction in the range of motion of the lower limb. Therefore, it is evident that lower limb muscle imbalance significantly impacts CAI. The core comprises the lumbo-pelvic-hip complex. An efficient core plays a vital role in maintaining normal length-tension relationships and force couples, which are essential for proper movement mechanics. It ensures optimal arthrokinematics, allowing joints to move smoothly and efficiently. A well-functioning core contributes to the overall efficiency of the entire kinetic chain, enabling better coordination during acceleration, deceleration, and dynamic stabilization. Additionally, it provides the necessary proximal stability for the proper movement of the extremities, supporting functional movement patterns and reducing the risk of injury. Lower limb muscle imbalance and core muscle weakness are significant contributors to CAI, impacting both stability and functional performance. Considering the study findings as an important part of the patient assessment will be used to customize the rehabilitation programs as per the patient's requirement and, thus, enhance positive outcomes.
